# Sustained Ability of a Natural Microbial Community to Remove Nitrate from Groundwater

**DOI:** 10.1111/gwat.13132

**Published:** 2021-09-22

**Authors:** Charles J. Paradis, John I. Miller, Ji‐Won Moon, Sarah J. Spencer, Lauren M. Lui, Joy D. Van Nostrand, Daliang Ning, Andrew D. Steen, Larry D. McKay, Adam P. Arkin, Jizhong Zhou, Eric J. Alm, Terry C. Hazen

**Affiliations:** ^1^ Department of Earth and Planetary Sciences University of Tennessee Knoxville TN; ^2^ Biosciences Division Oak Ridge National Laboratory Oak Ridge TN; ^3^ Bredesen Center University of Tennessee Knoxville TN; ^4^ Biological Engineering Department Massachusetts Institute of Technology Cambridge MA; ^5^ Environmental Genomics and Systems Biology Division Lawrence Berkeley National Laboratory Berkeley CA; ^6^ Institute for Environmental Genomics, Department of Microbiology and Plant Biology and School of Civil Engineering and Environmental Sciences, University of Oklahoma Norman OK; ^7^ Department of Microbiology University of Tennessee Knoxville TN; ^8^ Department of Bioengineering University of California Berkeley CA; ^9^ Department of Civil and Environmental Sciences University of Tennessee Knoxville TN; ^10^ Center for Environmental Biotechnology University of Tennessee Knoxville TN; ^11^ Institute for a Secure and Sustainable Environment University of Tennessee Knoxville TN

## Abstract

Microbial‐mediated nitrate removal from groundwater is widely recognized as the predominant mechanism for nitrate attenuation in contaminated aquifers and is largely dependent on the presence of a carbon‐bearing electron donor. The repeated exposure of a natural microbial community to an electron donor can result in the sustained ability of the community to remove nitrate; this phenomenon has been clearly demonstrated at the laboratory scale. However, in situ demonstrations of this ability are lacking. For this study, ethanol (electron donor) was repeatedly injected into a groundwater well (treatment) for six consecutive weeks to establish the sustained ability of a microbial community to remove nitrate. A second well (control) located upgradient was not injected with ethanol during this time. The treatment well demonstrated strong evidence of sustained ability as evident by ethanol, nitrate, and subsequent sulfate removal up to 21, 64, and 68%, respectively, as compared to the conservative tracer (bromide) upon consecutive exposures. Both wells were then monitored for six additional weeks under natural (no injection) conditions. During the final week, ethanol was injected into both treatment and control wells. The treatment well demonstrated sustained ability as evident by ethanol and nitrate removal up to 20 and 21%, respectively, as compared to bromide, whereas the control did not show strong evidence of nitrate removal (5% removal). Surprisingly, the treatment well did not indicate a sustained and selective enrichment of a microbial community. These results suggested that the predominant mechanism(s) of sustained ability likely exist at the enzymatic‐ and/or genetic‐levels. The results of this study demonstrated the in situ ability of a microbial community to remove nitrate can be sustained in the prolonged absence of an electron donor.

## Abstract 2

*Article Impact Statement*: Groundwater microbes sustain ability to remove nitrate in absence of carbon and energy source.

## Introduction

Natural microbial communities that can utilize nitrate as an electron acceptor are ubiquitous in groundwater and play a critical role in nitrate attenuation in contaminated aquifers (Rivett et al. 2008). The typical order in which electron acceptors are utilized is as follows: oxygen (O_2_), nitrate (NO_3_^−^), manganese (Mn(IV)), ferric iron (Fe(III)), and sulfate (SO_4_^2−^). The ability of these communities to reduce and effectively remove nitrate from groundwater is primarily limited by the availability of a suitable electron donor (Rivett et al. 2008). Ethanol has been shown to be an effective electron donor to stimulate nitrate removal in contaminated aquifers (Cardenas et al. 2008; Jin and Roden 2011; Vidal‐Gavilan et al. 2014) and is relatively labile, miscible, and easy to measure as compared to more complex donors (Fowdar et al. 2015). Prior exposure of a community to an electron donor can result in the sustained ability of the community to conduct specific donor‐acceptor reactions (Leahy and Colwell 1990; Kline et al. 2011). This phenomenon has been observed in the field based on characterization studies and has been demonstrated in the laboratory based on experimental studies (Koskella and Vos 2015).

For example, in the field, Pernthaler and Pernthaler (2005) observed the sustained ability of a marine microbial community in response to naturally fluctuating electron donor availability over the course of a single day. In the laboratory, Pernthaler et al. (2001) demonstrated that the sustained ability of marine isolates was dependent on the frequency of electron donor addition, for example, one species out‐competed the other during a single addition whereas the other species performed best during hourly additions. Leahy and Colwell (1990) summarized the predominant, yet inter‐related, mechanisms by which sustained ability can occur as follows: (1) induction and/or depression of specific enzymes; (2) genetic changes that result in new metabolic capabilities; and (3) selective enrichment of microbes able to conduct the donor‐acceptor reactions of interest. More recently and in the laboratory, Oh et al. (2013) demonstrated the inter‐related mechanisms of the sustained ability of a river sediment microbial community to utilize nitrate as an electron acceptor in response to exposures of an electron donor (benzalkonium chlorides); this resulted in both the selective enrichment of *Pseudomonas* species and genetic changes via benzalkonium chlorides‐related amino acid substitutions and horizontal gene transfer.

These observations, demonstrations, and mechanistic insights of the sustained ability of natural microbial communities conduct specific donor‐acceptor reactions are only a small fraction of those in the vast literature (Koskella and Vos 2015) yet they clearly illustrate the importance and highlight the current understanding of the topic. Nevertheless, there is a need to bridge the knowledge gap between field observations and laboratory demonstrations of sustained ability. Specifically, there is a need to design and conduct highly controlled field experiments with the proper controls to both demonstrate sustained ability and elucidate its mechanisms. The objectives of this study were as follows: (1) establish a natural microbial community able to utilize nitrate as an electron acceptor in groundwater; (2) determine how long sustained ability can last in the absence of a suitable electron donor; and (3) elucidate the microbial mechanism(s) responsible for sustained ability the community to remove nitrate.

## Materials and Methods

### Study Site

**Figure 1:**
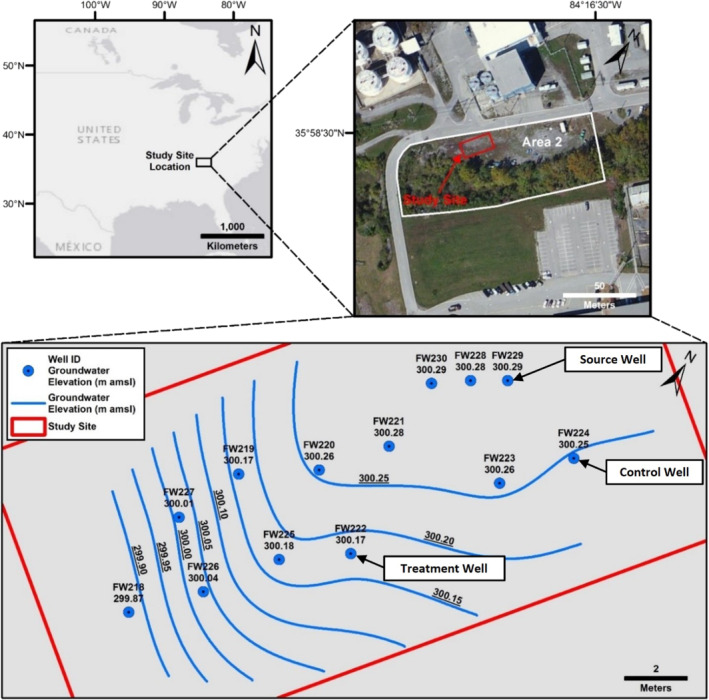
Plan‐view maps of the study site from Paradis et al. (2018), clockwise from upper left, country map showing study site location in the southeastern United States, area map showing study site location in Area 2 of the Oak Ridge Reserve, and study site map showing well locations (source well FW229, control well FW224, and treatment well FW222), groundwater elevations, and groundwater elevation iso‐contours. m amsl = meters above mean sea level.

The study site is in Area 2 of the Y‐12 S‐3 pond field site which is a part of the Oak Ridge Reservation (ORR) and in Oak Ridge, Tennessee, USA (Figure 1). The hydrogeology of the study site has been previously described (Watson et al. 2004; Paradis et al. 2016; Paradis et al. 2018). The subsurface consists of approximately 6 m of unconsolidated and heterogeneous materials comprised of silty and clayey fill underlain by undisturbed and clay‐rich weathered bedrock. The study site contains 13 monitoring wells (FW218 through FW230), two of which were used as test wells (FW222 [treatment well] and FW224 [control well]), and one of which was used as a source well (FW229) for groundwater injectate for the exposure tests (Figure 1). The test wells are constructed of 1.9‐cm inside diameter schedule‐80 polyvinyl chloride (PVC) pipe and are screened from 3.7 to 6.1 m below ground surface (mbgs). The test wells are screened within the fill materials and were vertically terminated at contact with the undisturbed weathered bedrock. The shallow groundwater aquifer is unconfined and the depth to groundwater is approximately 3.5 mbgs. The groundwater pH is circumneutral (pH ≈ 6.5 to 8.0) and dissolved oxygen (DO) is relatively low (DO ≈ 1–2 mg/L). Nitrate and sulfate concentrations are persistent due to the lack of a suitable electron donor and range from approximately 5 to 75 and 10 to 200 mg/L, respectively; the groundwater geochemistry has been previously described (Watson et al. 2004; Paradis et al. 2016; Paradis et al. 2018). The test wells (FW222 [treatment well] and FW224 [control well]) are separated by approximately 6 m of horizontal distance and are oriented up‐ and downgradient with respect to each other (Figure 1).

### Electron Donor Exposure Tests

**Figure 2:**
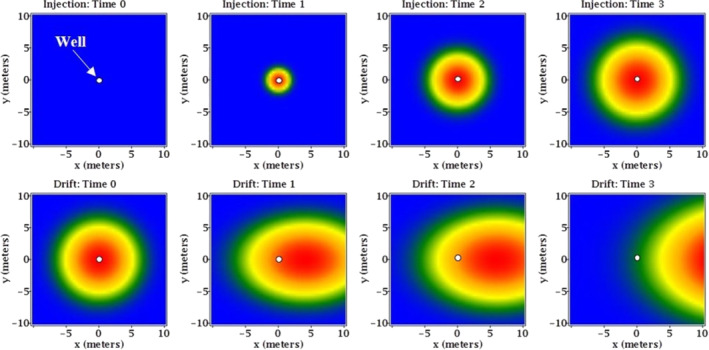
Conceptual model of a single‐well push‐pull test in plain view showing the forced‐flow injection (push) phase (top panel) and the natural‐flow drift (pull) phase (bottom panel); blue color represents the aquifer fluid, warmer colors represent the relative concentration of the injection fluid; natural groundwater flow is from left to right.

Electron donor exposure tests were conducted using the single‐well push‐pull test method (Istok 2013). During a push‐pull test, a volume of water which contains a known mass of one or more nonreactive and reactive tracers is injected into a single well under forced‐flow conditions; this is referred to as the push phase (Figure 2). The mixture of the injection fluid and aquifer fluid is then collected periodically from the same well under natural‐flow conditions; this is referred to as the pull or drift phase (Figure 2). The concentrations of the added tracers, reactants, and products are then plotted vs. the time elapsed to generate breakthrough curves. The breakthrough curves are then analyzed to characterize the mass transport mechanisms within the groundwater system, for example, advection, dispersion, sorption, and microbial‐mediated reactivity.

For this study, a volume of groundwater (5–40 L) was collected from upgradient well FW229 (Figure 1) using a peristaltic pump and stored in a plastic carboy. A mass of potassium bromide (KBr) (Sigma‐Aldrich) and ethanol (C_2_H_6_O) (Sigma‐Aldrich) was added to the injection solution and mixed by re‐circulation using a peristaltic pump for a target concentration of 200 mg/L bromide and 200 mg/L ethanol. Bromide was added as a nonreactive tracer whereas ethanol was added as a reactive tracer. The addition of ethanol (≈1400 mg/L) at the study site was previously shown to serve as a suitable electron donor to stimulate uranium removal (Paradis et al. 2016) whereas the focus of this study was to stimulate sustained nitrate removal. The injection solution was then injected into the test well (either treatment or control well), followed by a 20‐min resting period, and then periodically sampled over the course of 4 h. Immediately prior to, and after mixing of the injection solution, three samples were collected, filtered (0.2 μm filter), stored in 20‐mL scintillation vials without headspace, preserved at 4 °C, and promptly analyzed for bromide, nitrate, sulfate, and acetate by ion chromatography (Dionex ICS‐5000^+^) and for ethanol by gas chromatography (Agilent 6890). Acetate was previously shown to be the predominant metabolite of microbial‐mediated oxidation of ethanol under anaerobic conditions from sediments collected within Area 2 at the OR‐IFRC (Jin and Roden 2011). Three samples were also collected from the injection well (FW222 and/or FW224) immediately prior to injection and analyzed. The samples collected from the injection well (FW222 and/or FW224) immediately prior to injection were assumed to represent the aquifer fluid. The samples collected from the tracer‐added injection fluid immediately prior to injection were assumed to represent the injection fluid. The samples collected from the injection well (FW222 and/or FW224) after the injection were assumed to represent the mixture of the aquifer and injection fluids, that is, the extraction fluids.

A series of seven exposure tests were conducted in test well FW222 (treatment well) and one exposure test was conducted in test well FW224 (control well) (Table 1).

**Table 1:** Experimental Design of Electron Donor (EtOH) Exposure Tests and Microbial (DNA) Analysis for the Treatment Well (FW222) and Control Well (FW224)

Week	FW222 (Treatment Well)	FW224 (Control Well)
01	DNA analysis	DNA analysis
02	EtOH exposure 1	—
03	EtOH exposure 2	—
04	EtOH exposure 3, DNA analysis	DNA analysis
05	EtOH exposure 4	—
06	EtOH exposure 5	—
07	EtOH exposure 6, DNA analysis	DNA analysis
08	DNA analysis	DNA analysis
09	DNA analysis	DNA analysis
10	DNA analysis	DNA analysis
11	—	—
12	—	—
13	—	—
14	DNA Ana., EtOH Exp. 7, DNA Ana.	DNA Ana., EtOH Exp. 1, DNA Ana.

Ana. = analysis; DNA = 16S amplicon sequencing of rDNA from planktonic microbes; EtOH = ethanol; Exp. = exposure.

The treatment well was exposed to ethanol for six consecutive weeks (Weeks 2 through 7) followed by six consecutive weeks (Weeks 8 through 13) of no exposure to ethanol (Table 1). During this time, the control well was not exposed to ethanol and was subject only to natural hydrogeologic conditions. During Week 14, both the treatment and control wells were exposed to ethanol (Table 1). Both wells were sampled for DNA analysis throughout the 14‐week experiment (Table 1). The exposure tests allowed for comparing the effects of repeated exposure history (treatment well) vs. no exposure history (control well) in terms of microbial‐mediated removal of nitrate.

The breakthrough curves of bromide, ethanol, acetate, nitrate, and sulfate, were analyzed according to the general methodology of Paradis et al. (2019a, 2019b). In brief, three equations were used to characterize nonreactive transport, reactive transport, and the recovery factor of a reactive tracer, respectively, as follows:

(1) $$C_{e,1}=\left(C_{i,1}-C_{a,1}\right)e^{\mathrm{kt}}+C_{a,1}$$

where $C_{e,1}=$ concentration of nonreactive tracer in extraction fluid (L^3^/T); $C_{i,1}=$ concentration of nonreactive tracer in injection fluid (L^3^/T); $C_{a,1}=$ concentration of nonreactive tracer in aquifer fluid (L^3^/T); k= first‐order dilution rate (1/T); t= time elapsed (T); and

(2) $$C_{e,2}^{*}=\left(\frac{C_{e,1}-C_{a,1}}{C_{i,1}-C_{a,1}}\right)\left(C_{i,2}-C_{a,2}\right)+C_{a,2}$$

where $C_{e,2}^{*}=$ expected concentration of reactive tracer in extraction fluid due to dilution (L^3^/T); $C_{i,2}=$ concentration of reactive tracer in injection fluid (L^3^/T); $C_{a,2}=$ concentration of nonreactive tracer in aquifer fluid (L^3^/T); and

(3) $$\mathrm{RF}=\frac{\int_{t_{o}}^{t}C_{e,2}\left(t\right)\mathrm{dt}}{\int_{t_{o}}^{t}C_{e,2}^{*}\left(t\right)\mathrm{dt}}$$

where RF= recovery factor (dimensionless); $C_{e,2}=$ measured concentration of reactive tracer in extraction fluid (L^3^/T).

Equation (1) describes the dilution of the finite volume of injection fluid with respect to the nearly infinite volume of aquifer fluid where the first‐order dilution rate (k) is proportional to the rate of groundwater flow through the well and its surrounding aquifer material. Equation (2) describes the expected concentration of a reactive tracer in the extraction fluid due to dilution of the injection fluid where any difference between its expected concentration ($C_{e,2}^{*}$) and its measured concentration ($C_{e,2}$) can be attributed to one or more reactive processes, for example, microbial‐mediated reactivity. Equation (3) describes the ratio of the measured mass recovery of a tracer as compared its expected mass recovery when accounting for dilution. For example, a recovery factor (RF) greater than one indicates a net addition of the tracer to the aqueous phase whereas an RF less than one indicates a net removal of the tracer from the aqueous phase and an RF equal to one indicates no change. Equation (3) must be evaluated using numerical integration methods because the breakthrough curve data is both discrete and its underlying continuous function is unknown. For this study, Equation (3) was evaluated using the mid‐point, trapezoid, and Simpson's techniques and the average RF plus or minus its standard error was reported.

### Microbial Community Structure

The test wells were sampled for microbial community structure according to the general methodology of Smith et al. (2015). A volume of groundwater (5–10 L) was collected from the wells immediately prior to and following the exposure tests (Table 1). The groundwater was filtered, in series, through a 10‐μm filter (to remove particulate matter) and a 0.2‐μm filter (to capture microbial biomass) and preserved at −80 °C. Microbial DNA was extracted from the 0.2‐μm filter using a modified Miller method (Miller et al. 1999; Hazen et al. 2010; Smith et al. 2015) and shipped to the Institute for Environmental Genomics (Norman, OK, USA) for analysis of microbial DNA.

Extracted DNA was amplified as described in Wu et al. (2015). DNA was polymerase chain reaction (PCR) amplified using a two‐step PCR. In the first step, 16S rDNA was amplified for 10 cycles using primers 515F and 806R. In the second step, product from the first step was amplified for an additional 20 cycles using primers containing spacers to increase base diversity, barcodes, Illumina adaptor and sequencing primers, and the target primers, 515F and 806R. Amplification efficiency was evaluated by agarose gel electrophoresis. PCR products were pooled in equal molality and purified. Sequencing libraries were prepared according to the MiSeq™ Reagent Kit Preparation Guide (Illumina, San Diego, CA, USA) (Caporaso et al. 2012). Sequencing was performed for 251, 12, and 251 cycles for forward, index, and reverse reads, respectively, on an Illumina MiSeq using a 500‐cycle v2 MiSeq reagent cartridge.

The resulting DNA sequences were analyzed according to the general methodology of Techtmann et al. (2015). DNA sequences were analyzed using the QIIME version 1.8.0‐dev pipeline (Caporaso et al. 2012) and paired‐end raw reads were joined using fastq‐join (Aronesty 2015). The joined sequences were demultiplexed and quality filtered in QIIME to remove reads with phred scores below 20. Chimera detection was then performed on joined reads using UCHIME (Edgar 2010; Edgar et al. 2011). Joined, quality‐filtered and chimera‐checked sequences were deposited at MG‐RAST. Sequences were clustered into operational taxonomic units (OTUs, 97% similarity) with UCLUST (Edgar 2010) using the open reference clustering protocol. The resulting representative sequences were aligned using PyNAST (Caporaso et al. 2010) and given a taxonomic assignment using Ribosomal database project (RDP) (Wang et al. 2007) retrained with the May 2013 Greengenes release. The resulting OTU table was filtered to keep OTUs that were present at greater than 0.005%, and then rarified to 13,753 sequences per sample (the minimum number of remaining sequences in the samples).

To test the hypothesis that exposure to ethanol influenced community structure, nonmetric multi‐dimensional scaling (NMDS) and hierarchical clustering analysis (HCA) were performed. A Bray‐Curtis (BC) dissimilarity matrix was constructed using the scipy.spatial.distance methods from the SciPy library (Jones et al. 2001) in Python (Rossum 2017) and used as input for NMDS and HCA. NMDS was performed using the sklearn.manifold methods from the Scikit‐learn library (Pedregosa et al. 2011). At two dimensions, stress was approximately 4, while at three dimensions and higher, stress was approximately 0.5. The goal of NMDS is to provide a low‐dimensional visualization of the similarity of the microbial communities; therefore, three dimensions were chosen as the fewest dimensions with the lowest stress value. HCA was performed with the scipy.cluster.hierarchy methods using the average linkage method. The number of dimensions was increased starting from two to identify the minimum number of dimensions necessary to achieve a reasonable stress value. A breakpoint was identified at three dimensions, above which ordination stress did not decrease substantially.

## Results and Discussion

### Electron Donor Exposure Tests

**Figure 3:**
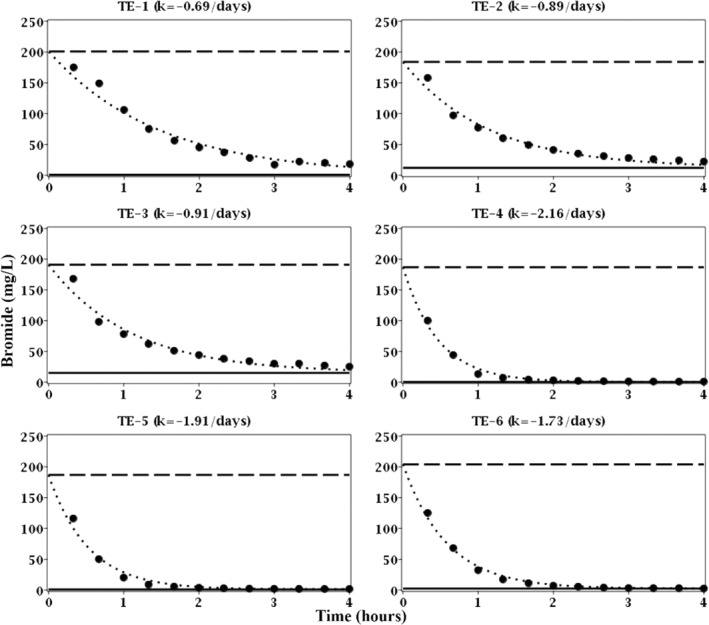
Breakthrough curves of bromide (nonreactive tracer) for treatment exposures 1 through 6 (TE‐1 through TE‐6) in well FW222. Solid circles (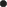) are concentrations of bromide in the extraction fluid; dashed line (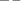) is the concentration of bromide in the injection fluid; solid line (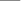) is the concentration of bromide in the aquifer fluid or the lower detection limit; and dotted line (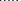) is the best fit of the first‐order dilution rate (*k*).

The breakthrough curves of bromide in the treatment well (FW222) during the six consecutive weeks of ethanol exposure demonstrated first‐order dilution rates (Equation 1) ranging from −0.69 to −2.16/days (Figure 3). The dilution rates during the latter 3 weeks (TE‐4, TE‐5, and TE‐6 in Figure 3) were substantially greater then observed during the first 3 weeks (TE‐1, TE‐2, and TE‐3 in Figure 3). These results indicated that the rate of groundwater flow through the treatment well and its surrounding aquifer material was transient as opposed to steady state. The transient behavior of groundwater flow was not surprising when considering that the aquifer is unconfined and the depth to groundwater is relatively shallow (approximately 3.5 mbgs); these hydrogeologic characteristics make the aquifer particularly sensitive to recharge and discharge events. It should be noted that a series of precipitation events occurred during the latter 3 weeks (TE‐4, TE‐5, and TE‐6 in Figure 3) and that the water table level rose during this time; these recharge events may have been the cause of the observed increase in the dilution rate as no pumping wells within the vicinity of the study site were in operation.

**Figure 4:**
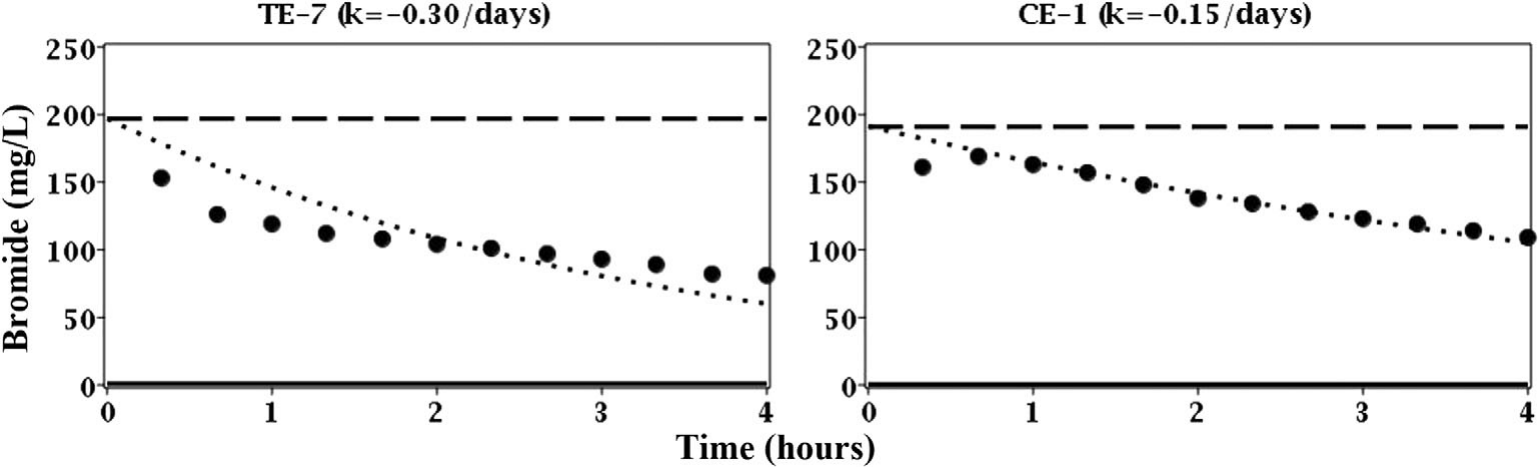
Breakthrough curves of bromide (nonreactive tracer) for treatment exposure 7 (TE‐7) well FW222 and control exposure 1 (CE‐1) in well FW224. Solid circles (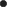) are concentrations of bromide in the extraction fluid; dashed line (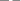) is the concentration of bromide in the injection fluid; solid line (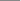) is the concentration of bromide in the aquifer fluid or the lower detection limit; and dotted line (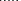) is the best fit of the first‐order dilution rate (*k*).

The breakthrough curves of bromide in the treatment and control wells during the final week of ethanol exposure also demonstrated first‐order dilution rates (Figure 4). However, these rates were relatively low (−0.15 to −0.30/days) as compared to the first 6 weeks (Figure 3) and further indicated the transient behavior of groundwater flow. Nevertheless, the rates of groundwater flow during the final week of ethanol exposure in both treatment and control wells were notably similar as evident by dilution rates within a factor of two (Figure 4). It must be noted that the breakthrough curves bromide (Figures 3 and 4) were interpreted to represent nonreactive dilution between the injection and aquifer fluids.

**Figure 5:**
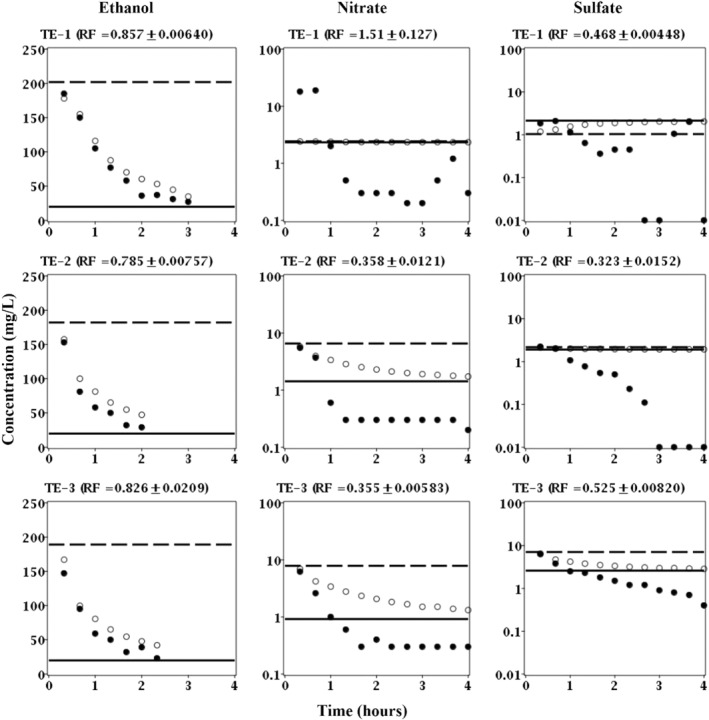
Breakthrough curves of ethanol, nitrate, and sulfate for treatment exposures 1 through 3 (TE‐1 through TE‐3) in well FW222. Solid circles (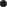) are measured concentrations in the extraction fluid; open circles (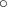) are expected concentrations in the extraction fluid based on bromide (nonreactive tracer); dashed line (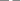) is the concentration in the injection fluid; and solid line (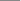) is the concentration in the aquifer fluid or the lower detection limit.

The breakthrough curves of ethanol, nitrate, and sulfate for exposure one in the treatment well (FW222) did not demonstrate concomitant removal of ethanol and nitrate or sulfate as evident by the lack of clear and convincing trends in the data or recovery factors (TE‐1 in Figure 5). These results suggested that the natural microbial community was not readily able to utilize ethanol and nitrate. However, the breakthrough curves for exposures two and three did demonstrate concomitant ethanol and nitrate removal and subsequent sulfate removal as evident by lower than expected concentrations; nitrate and sulfate concentrations actually fell below even that of the aquifer fluid (TE‐2 and TE‐3 in Figure 5). Moreover, the recovery factors for ethanol, nitrate, and sulfate were consistently less than one, 0.826 to 0.785, 0.358 to 0.355, and 0.525 to 0.323, respectively (TE‐2 and TE‐3 in Figure 5). These recovery factors indicated that up to 21, 64, and 68% of ethanol, nitrate, and sulfate, respectively, was removed as compared to bromide.

Microbial‐mediated oxidation of ethanol to acetate and reduction of nitrate and sulfate has been well documented at the study site (Wu et al. 2006; Wu et al. 2007) and abroad (Feris et al. 2008; Vidal‐Gavilan et al. 2014; Rodriguez‐Escales et al. 2016). Moreover, the relative increase in microbial activity during subsequent exposures to ethanol, that is, sustained ability, was expected based on previous studies (Kline et al. 2011). Acetate production was observed for exposures one, two, and three as evident by recovery factors greater than one (data not shown). However, given that acetate is an intermediate byproduct of ethanol reduction and can serve as an electron donor for further reduction its temporal behavior is somewhat difficult to interpret beyond evidence of ethanol oxidation.

The rate of groundwater flow was so high for exposures four, five, and six (TE‐4, TE‐5, and TE‐6 in Figure 3) that the concentration of ethanol was diluted to below the method detection limit (20 mg/L) within the first hour and therefore only two or three data points were available for analysis (data not shown).

**Figure 6:**
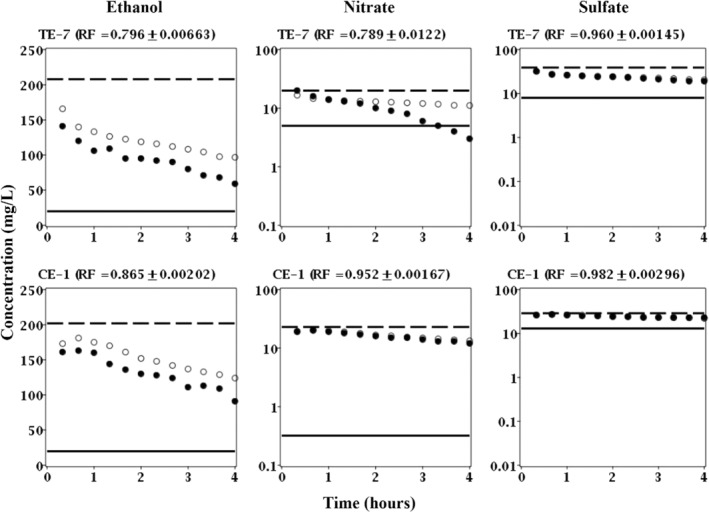
Breakthrough curves of ethanol, nitrate, and sulfate for treatment exposure 7 (TE‐7) in well FW222 and control exposure 1 (CE‐1) in well FW224; both occurring in Week 14 (Table 1). Solid circles (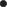) are measured concentrations in the extraction fluid; open circles (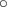) are expected concentrations in the extraction fluid based on bromide (nonreactive tracer); dashed line (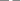) is the concentration in the injection fluid; and solid line (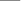) is the concentration in the aquifer fluid or the lower detection limit.

The breakthrough curves of ethanol, nitrate, and sulfate for exposure seven in the treatment well (FW222) demonstrated concomitant ethanol and nitrate removal as evident by lower than expected concentrations; again, nitrate concentrations actually fell below even that of the aquifer fluid (TE‐7 in Figure 6). Moreover, the recovery factors for both ethanol and nitrate were much less than one, 0.796 and 0.789, respectively (TE‐7 in Figure 6). These recovery factors indicated that up to 20 and 21% of ethanol and nitrate, respectively, was removed as compared to bromide.

In contrast, the breakthrough curves for exposure one in the control well (CE‐1 in Figure 6) and exposure one in the treatment well (TE‐1 in Figure 5) were comparable in that some ethanol removal was observed but not nitrate; nitrate concentrations in the control well were nearly identical to those expected due to dilution (CE‐1 in Figure 6). Moreover, the recovery factor for nitrate was nearly equal to one, 0.952 to be exact (CE‐1 in Figure 6). This recovery indicated that only up to 5% of nitrate was removed as compared to bromide.

Interestingly, the recovery factor for ethanol was less than one, 0.865 to be exact (CE‐1 in Figure 6). Moreover, acetate production was also observed, although substantially less as compared to the treatment well (data not shown). One explanation for the apparent removal of ethanol but not nitrate in the first exposure of the control well (CE‐1 in Figure 6) and the first exposure of the treatment well (TE‐1 in Figure 5) is the presence of oxygen as a higher energy yielding electron acceptor. For example, it is likely that oxygen was introduced to the injection fluid during the aboveground mixing of bromide and ethanol; unfortunately, oxygen was not measured to confirm this. However, it is possible that aerobic respiration of ethanol occurred rapidly and prior to the onset of anaerobic conditions when nitrate would be the next highest energy yielding electron acceptor. Another, simpler, explanation for the apparent removal of ethanol but not nitrate is that ethanol may have preferentially volatized and/or adsorbed to the aquifer materials as compared to bromide; these abiotic mass transport processes would result in a recovery factor of ethanol that was less than one but fail to explain any observed production of acetate.

Overall, these results strongly suggested that the treatment well sustained its ability for nitrate removal even in the absence of ethanol for up to 6 weeks. It is conceivable that the duration of sustained ability could have lasted much longer and therefore additional in situ studies are needed to constrain an upper limit on the duration of this phenomenon.

### Microbial Community Structure

**Figure 7:**
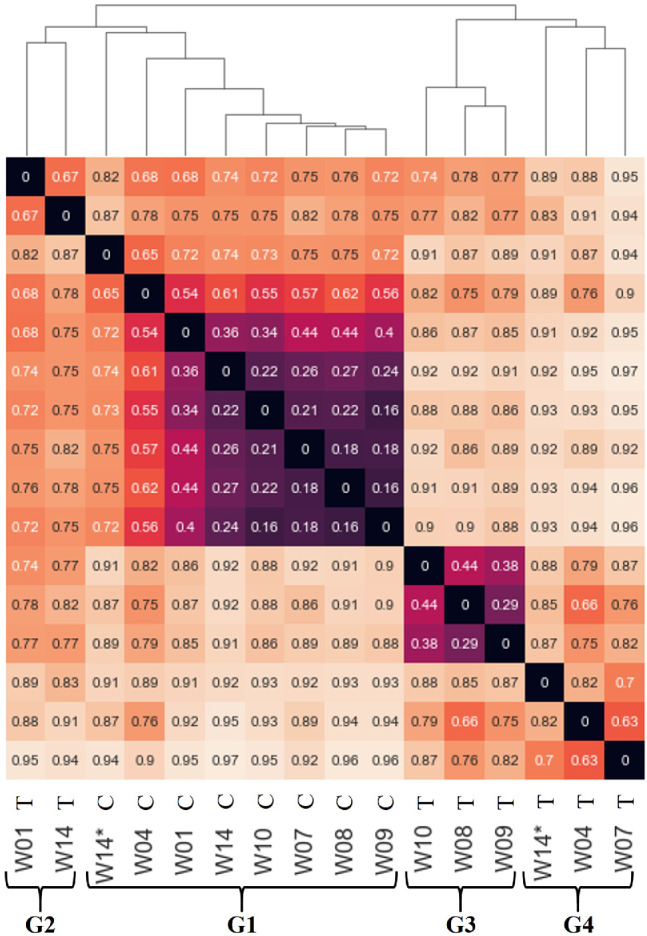
Heat map and hierarchical clustering analysis (HCA) of Bray‐Curtis dissimilarity between microbial communities. Samples are labeled by week number (W01–W14) and site (control C or treatment T); * indicates community after final ethanol exposure. G1, G2, G3, and G4 indicate distinct groups. The heat map is a symmetric matrix (*x* and *y* axes are the same) showing the pair‐wise Bray‐Curtis dissimilarity between microbial communities (0 indicates identical community composition, 1 indicating no overlap in composition). HCA begins by clustering the two communities that are the most similar (i.e., smallest Bray‐Curtis dissimilarity value) to each other into a single group. This process is repeated until all communities are placed in a single group. The dendrogram illustrates the order in which communities are clustered, and the height of the lines represents the dissimilarity between the communities (or clusters) at that step. G1 consisted entirely of communities from the control well, whereas G2, G3, and G4 consisted entirely of communities from the treatment well. G2 is comprised of the pre‐ethanol exposure community (W01) and 7 weeks after cessation of ethanol exposure (W14). G3 is comprised of communities following 6 weeks of ethanol exposure, and G4 is comprised of communities during initial or final ethanol exposure.

**Figure 8:**
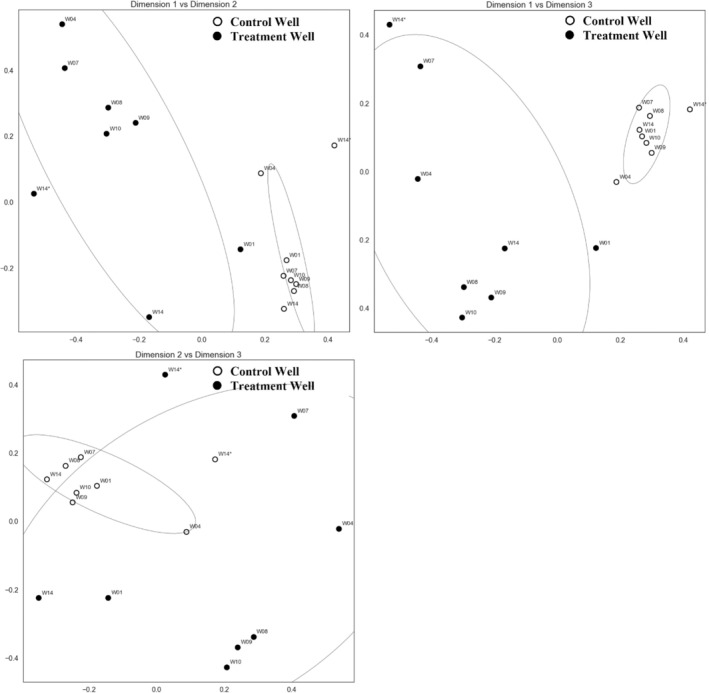
Nonmetric multidimensional scaling (NMDS) of Bray‐Curtis dissimilarity between microbial communities. Markers indicate control or treatment well, and labels show week of sampling (W01 through W14*); * indicates community after final ethanol exposure. Ellipses illustrate the 95% confidence ellipse around the control and treatment community samples. NMDS was performed to three dimensions to reduce stress. The communities from the control wells were more closely related to each other than the communities from the treatment well, which suggests that ethanol exposure influences community composition.

Pair‐wise BC dissimilarity between microbial communities was used to identify changes in community composition at the control and treatment wells. HCA and NMDS analysis of BC was performed to further assess the similarity of the natural microbial communities (Figures 7 and 8). HCA indicated that communities clustered into four distinct groups: G1, G2, G3, and G4 (Figure 7). Group 1 (G1) consisted entirely of communities from the control well, whereas Groups 2, 3, and 4 consisted entirely of communities from the treatment well. Notably, within the control well (G1), the community composition following ethanol exposure (W14*) was most dissimilar as indicated by the dendrogram (Figure 7). NMDS analysis further illustrated that the microbial community at the control well showed relatively little variability over time, while there were large changes in the composition of the community at the treatment well (Figure 8).

Group 2 (G2) consisted of the treatment well at Week 1 (W01) and Week 14 prior to ethanol exposure (W14), which were more similar to each other (BC = 0.67) than to communities at other time points (BC ≥0.68) across both treatment and control (Figures 7 and 8). Despite the shift of the microbial community at the treatment site back toward its initial composition (W01), the breakthrough curves suggested that the W14 community sustained its ability for ethanol‐induced nitrate removal (Figures 5 and 6). Group 3 (G3) consisted of communities from the treatment well at Weeks 8, 9, and 10, and Group 4 (G4) consisted of communities from the same well at Weeks 4, 7, and Week 14* (Figure 7). G3 communities coincided with the 6‐week period following ethanol exposure, whereas G4 included communities during the initial 6‐week period of ethanol exposure (W04, W07) and following final ethanol exposure (W14*) (Figure 7 and Table 1). It is interesting that when ethanol exposure stopped after Week 7, the community composition shifted back toward the initial community composition, which is evident from the similarity of W01 and W14 in G2. This result was particularly interesting considering the sustained ability for ethanol‐induced nitrate removal observed by the community at Week 14 (Figures 5 and 6), and that the composition rapidly shifted back toward the composition during initial ethanol exposure (W14*). There are two possible explanations for rapid nitrate removal at the treatment site during Week 14: (1) prior ethanol exposure enriched the microbial community for microbes that consume nitrate, and these changes were persistent through the end of this experiment; or (2) prior ethanol exposure induced genetic adaptations in members of the microbial community that favor nitrate consumption. It is also possible that the sessile microbial community was readily able to utilize ethanol, but without sediment samples this could not be tested. The extent to which each of these mechanisms contributes to nitrate removal remains unknown.

**Figure 9:**
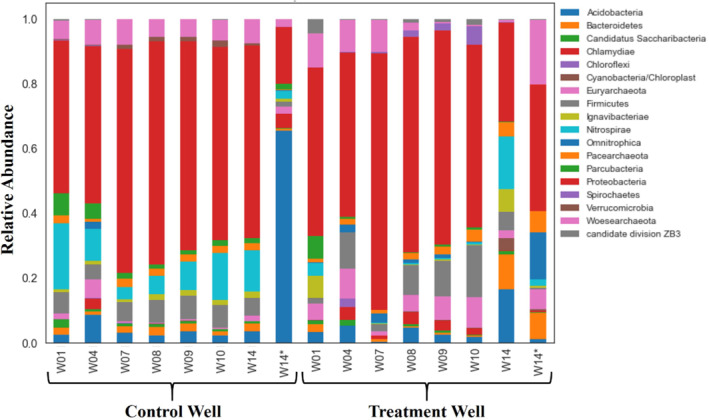
Relative abundance of microbial taxa at the phylum level during the 14‐week experiment (W01 through W14) for the control and treatment wells; * indicates community after final ethanol exposure. Each column represents an entire community, and each of the color‐coded bars represents a single taxon in that community. Abundance is quantified as the proportion of DNA sequences assigned to that taxon.

Relative abundance analysis was conducted to assess the shifts in particular taxa at the level of phylum (Figure 9). The microbial community of the control well was dominated by *Proteobacteria* for Weeks 1 through the beginning of 14 but showed considerable variability (Figure 9). The relative abundance of other taxa in the control well, such as *Nitrospirae*, *Firmicutes*, and *Woesearchaeota* were also notable for Weeks 1 through the beginning of 14 and showed considerable variability (Figure 9). During this time, the control well was not exposed to ethanol (Table 1). Therefore, the temporal changes in taxa in the control well for Weeks 1 through 14 were representative of natural biogeochemical conditions. The high relative abundance and temporal variability of *Proteobacteria*, *Nitrospirae*, and *Firmicutes* under natural biogeochemical conditions was expected based on a recent study at the ORR by King et al. (2017). King et al. (2017) demonstrated similar results from in situ aboveground bioreactors and noted that such taxa are associated with low DO and/or representative of nitrate reducers. Both low DO and the presence of nitrate are characteristic of the dissolved‐phase chemistry at the study site (Paradis et al. 2016). The control well was exposed to ethanol during the middle of Week 14 (W14) and sampled for microbial community structure at the end of Week 14 (W14*) (Table 1). After exposure to ethanol (W14*), *Acidobacteria* substantially increased in relative abundance, replacing *Proteobacteria* as the dominant phylum (Figure 9). These results differ from previous studies at the ORR which showed increases of *Proteobacteria* and decreases of *Acidobacteria* after exposure to ethanol (Spain et al. 2007; Cardenas et al. 2008). However, those studies characterized the microbial communities associated with sediment (sessile) and after prolonged (3 weeks to 2 years) exposures of ethanol (Spain et al. 2007; Cardenas et al. 2008) whereas this study characterized microbial communities associated with groundwater (planktonic) and after a brief (less than 4 h) exposure of ethanol. It is possible that the sessile microbial community changed in a manner consistent with previous studies, but this is not known due to lack of sediment samples. It is also possible that duration of exposure to ethanol, that is, prolonged vs. brief, had a notable effect on the relative abundance of taxa as previously demonstrated by Pernthaler et al. (2001). Nevertheless, these results demonstrated that the planktonic microbial community in the control well was relatively stable under natural conditions but rapidly changed after exposure to ethanol.

The treatment well was dominated by *Proteobacteria* for Weeks 1 through 10 but varied considerably more than the control well (Figure 9). The relative abundance of other taxa in the treatment well, such as *Firmicutes* and *Woesearchaeota* were also notable for Weeks 1 through 10 and showed considerable variability (Figure 9). Compared to the control well during this time, the community in the treatment well by Week 10 was notably different than Week 1 (Figure 9). A notable change in the community in the treatment well was expected because by Week 10 the treatment had been exposed to six consecutive weeks of ethanol whereas the exposure control had not been exposed to ethanol (Table 1). By the beginning of Week 14, the treatment well had been exposed to ethanol for six consecutive weeks followed by six consecutive weeks without exposure to ethanol (Table 1). As compared to the control well, the community in the treatment well by Week 14 was notably different than Week 1 (Figure 9). Therefore, if the microbial community in the treatment was able, and sustained its ability for, ethanol‐induced removal of nitrate, which the breakthrough curves strongly suggested (Figure 6), then the community at the beginning of Week 14 (W14) may be representative of a sustained community (Figure 9). Likewise, if the microbial community in the control well lacked the sustained ability for ethanol‐induced removal of nitrate, which the breakthrough curves strongly suggested (Figure 6), then the community at the beginning of Week 14 (W14) may be representative of a nonable community (Figure 9). The relative abundance of taxa in the treatment well after its final exposure to ethanol (W14*) was notably different than before its final exposure to ethanol (W14) as indicated by the increase of *Woesearchaeota* and decrease of *Nitrospirae* (Figure 9). These results demonstrated that the microbial community in the exposure treatment changed upon exposure to ethanol and sustained a level of ability in the absence of exposure to ethanol. As previously noted, it is also possible that genetic changes, rather than persistent changes to the community structure, were the primary mechanism that allowed the treatment well to respond rapidly to ethanol exposure (W14*). Therefore, future in situ studies of sustained ability should attempt to characterize the sessile community as well as investigate the genetic changes to ethanol exposure.

## Conclusions

The objectives of this study were to establish an in situ natural microbial community able to remove nitrate from groundwater via the addition of an electron donor and then determine how long this ability could be sustained in the absence of the electron donor and elucidate the microbial mechanism(s) responsible for this ability. The results of this study strongly suggested that the in situ ability of a natural microbial community to remove nitrate from groundwater can be sustained in the prolonged absence of an electron donor; in this case, at least 6 weeks in the absence of ethanol. However, this ability was not revealed in the experiment by a sustained and selected enrichment of a planktonic microbial community based on 16S rDNA. Therefore, it is possible that such a microbial community may be present in the sessile state or that the predominant mechanism(s) of this ability exist at the enzymatic‐ and/or genetic‐levels. Nevertheless, this study demonstrated that the exposure history of groundwater to an electron donor can play an important role in the removal of nitrate.

## Authors' Note

The authors do not have any conflicts of interest or financial disclosures to report.

## References

[gwat13132-bib-0001] Aronesty, E. 2013. ea‐utils: Command‐line tools for processing biological sequencing data. The open bioinformatics journal 7, no. 1: 845.

[gwat13132-bib-0002] Caporaso, J.G. , C.L. Lauber , W.A. Walters , D. Berg‐Lyons , J. Huntley , N. Fierer , S.M. Owens , J. Betley , L. Fraser , M. Bauer , N. Gormley , J.A. Gilbert , G. Smith , and R. Knight . 2012. Ultra‐high‐throughput microbial community analysis on the Illumina HiSeq and MiSeq platforms. ISME Journal 6, no. 8: 1621–1624. 22402401 10.1038/ismej.2012.8 PMC3400413

[gwat13132-bib-0003] Caporaso, J.G. , K. Bittinger , F.D. Bushman , T.Z. DeSantis , G.L. Andersen , and R. Knight . 2010. PyNAST: A flexible tool for aligning sequences to a template alignment. Bioinformatics 26, no. 2: 266–267. 19914921 10.1093/bioinformatics/btp636 PMC2804299

[gwat13132-bib-0004] Cardenas, E. , W.M. Wu , M.B. Leigh , J. Carley , S. Carroll , T. Gentry , J. Luo , D. Watson , B. Gu , M. Ginder‐Vogel , P.K. Kitanidis , P.M. Jardine , J. Zhou , C.S. Criddle , T.L. Marsh , and J.A. Tiedje . 2008. Microbial communities in contaminated sediments, associated with bioremediation of uranium to submicromolar levels. Applied and Environmental Microbiology 74, no. 12: 3718–3729. 18456853 10.1128/AEM.02308-07 PMC2446554

[gwat13132-bib-0005] Edgar, R.C. , B.J. Haas , J.C. Clemente , C. Quince , and R. Knight . 2011. UCHIME improves sensitivity and speed of chimera detection. Bioinformatics 27, no. 16: 2194–2200. 21700674 10.1093/bioinformatics/btr381 PMC3150044

[gwat13132-bib-0006] Edgar, R.C. 2010. Search and clustering orders of magnitude faster than BLAST. Bioinformatics 26, no. 19: 2460–2461. 20709691 10.1093/bioinformatics/btq461

[gwat13132-bib-0007] Feris, K. , D. Mackay , N. de Sieyes , I. Chakraborty , M. Einarson , K. Hristova , and K. Scow . 2008. Effect of ethanol on microbial community structure and function during natural attenuation of benzene, toluene, and o‐xylene in a sulfate‐reducing aquifer. Environmental Science & Technology 42, no. 7: 2289–2294. 18504955 10.1021/es702603q

[gwat13132-bib-0008] Fowdar, H.S. , B.E. Hatt , P. Breen , P.L.M. Cook , and A. Deletic . 2015. Evaluation of sustainable electron donors for nitrate removal in different water media. Water Research 85: 487–496. 26379204 10.1016/j.watres.2015.08.052

[gwat13132-bib-0009] Hazen, T.C. , E.A. Dubinsky , T.Z. DeSantis , G.L. Andersen , Y.M. Piceno , N. Singh , J.K. Jansson , A. Probst , S.E. Borglin , J.L. Fortney , W.T. Stringfellow , M. Bill , M.E. Conrad , L.M. Tom , K.L. Chavarria , T.R. Alusi , R. Lamendella , D.C. Joyner , C. Spier , J. Baelum , M. Auer , M.L. Zemla , R. Chakraborty , E.L. Sonnenthal , P. D'Haeseleer , H.Y.N. Holman , S. Osman , Z.M. Lu , J.D. Van Nostrand , Y. Deng , J.Z. Zhou , and O.U. Mason . 2010. Deep‐sea oil plume enriches indigenous oil‐degrading bacteria. Science 330, no. 6001: 204–208. 20736401 10.1126/science.1195979

[gwat13132-bib-0010] Istok, J.D. 2013. Push‐Pull Tests for Site Characterization, 83. Heidelberg, Germany; New York; Dordrecht, The Netherlands; London, UK: Springer.

[gwat13132-bib-0011] Jin, Q.S. , and E.E. Roden . 2011. Microbial physiology‐based model of ethanol metabolism in subsurface sediments. Journal of Contaminant Hydrology 125, no. 1–4: 1–12. 21652106 10.1016/j.jconhyd.2011.04.002

[gwat13132-bib-0012] Jones, E. , T. Oliphant , and P. Peterson . 2001. http://www.scipy.org/ SciPy: Open Source Scientific Tools for Python.

[gwat13132-bib-0013] King, A.J. , S.P. Preheim , K.L. Bailey , M.S. Robeson , T.R. Chowdhury , B.R. Crable , R.A. Hurt , T. Mehlhorn , K.A. Lowe , T.J. Phelps , A.V. Palumbo , C.C. Brandt , S.D. Brown , M. Podar , P. Zhang , W.A. Lancaster , F. Poole , D.B. Watson , M.W. Fields , J.M. Chandonia , E.J. Alm , J.Z. Zhou , M.W.W. Adams , T.C. Hazen , A.P. Arkin , and D.A. Elias . 2017. Temporal dynamics of in‐field bioreactor populations reflect the groundwater system and respond predictably to perturbation. Environmental Science & Technology 51, no. 5: 2879–2889. 28112946 10.1021/acs.est.6b04751

[gwat13132-bib-0014] Kline, K.R. , J.F. Clark , L. Rastegarzadeh , Y.M. Nelson , and D.M. Mackay . 2011. Importance of exposure history when using single well push‐pull tests to quantify in situ ethanol biodegradation rates. Ground Water Monitoring and Remediation 31, no. 3: 103–110.

[gwat13132-bib-0015] Koskella, B. , and M. Vos . 2015. Adaptation in natural microbial populations. In Annual Review of Ecology, Evolution, and Systematics, Vol. 46, ed. D.J. Futuyma , 503–522. 10.1146/annurev-ecolsys-112414-054458

[gwat13132-bib-0016] Leahy, J.G. , and R.R. Colwell . 1990. Microbial‐degradation of hydrocarbons in the environment. Microbiological Reviews 54, no. 3: 305–315. 2215423 10.1128/mr.54.3.305-315.1990 PMC372779

[gwat13132-bib-0017] Miller, D.N. , J.E. Bryant , E.L. Madsen , and W.C. Ghiorse . 1999. Evaluation and optimization of DNA extraction and purification procedures for soil and sediment samples. Applied and Environmental Microbiology 65, no. 11: 4715–4724. 10543776 10.1128/aem.65.11.4715-4724.1999 PMC91634

[gwat13132-bib-0018] Oh, S. , M. Tandukar , S.G. Pavlostathis , P.S.G. Chain , and K.T. Konstantinidis . 2013. Microbial community adaptation to quaternary ammonium biocides as revealed by metagenomics. Environmental Microbiology 15, no. 10: 2850–2864. 23731340 10.1111/1462-2920.12154

[gwat13132-bib-0019] Paradis, C.J. , E.R. Dixon , L.M. Lui , A.P. Arkin , J.C. Parker , J.D. Istok , E. Perfect , L.D. McKay , and T.C. Hazen . 2019a. Improved method for estimating reaction rates during push‐pull tests. Groundwater 57, no. 2: 292–302. 10.1111/gwat.12770 PMC7379995 29656383

[gwat13132-bib-0020] Paradis, C.J. , L.D. McKay , E. Perfect , J.D. Istok , and T.C. Hazen . 2019b. Correction: Push‐pull tests for estimating effective porosity: Expanded analytical solution and in situ application. Hydrogeology Journal 27, no. 1: 437–439.

[gwat13132-bib-0021] Paradis, C.J. , L.D. McKay , E. Perfect , J.D. Istok , and T.C. Hazen . 2018. Push‐pull tests for estimating effective porosity: Expanded analytical solution and in situ application. Hydrogeology Journal 26, no. 2: 381–393.

[gwat13132-bib-0022] Paradis, C.J. , S. Jagadamma , D.B. Watson , L.D. McKay , T.C. Hazen , M. Park , and J.D. Istok . 2016. In situ mobility of uranium in the presence of nitrate following sulfate‐reducing conditions. Journal of Contaminant Hydrology 187: 55–64. 26897652 10.1016/j.jconhyd.2016.02.002

[gwat13132-bib-0023] Pedregosa, F. , G. Varoquaux , A. Gramfort , V. Michel , B. Thirion , O. Grisel , M. Blondel , P. Prettenhofer , R. Weiss , V. Dubourg , J. Vanderplas , A. Passos , D. Cournapeau , M. Brucher , M. Perrot , and E. Duchesnay . 2011. Scikit‐learn: Machine learning in Python. Journal of Machine Learning Research 12: 2825–2830.

[gwat13132-bib-0024] Pernthaler, A. , and J. Pernthaler . 2005. Diurnal variation of cell proliferation in three bacterial taxa from coastal North Sea waters. Applied and Environmental Microbiology 71, no. 8: 4638–4644. 16085858 10.1128/AEM.71.8.4638-4644.2005 PMC1183367

[gwat13132-bib-0025] Pernthaler, A. , J. Pernthaler , H. Eilers , and R. Amann . 2001. Growth patterns of two marine isolates: Adaptations to substrate patchiness? Applied and Environmental Microbiology 67, no. 9: 4077–4083. 11526008 10.1128/AEM.67.9.4077-4083.2001 PMC93132

[gwat13132-bib-0026] Rivett, M.O. , S.R. Buss , P. Morgan , J.W.N. Smith , and C.D. Bemment . 2008. Nitrate attenuation in groundwater: A review of biogeochemical controlling processes. Water Research 42, no. 16: 4215–4232. 18721996 10.1016/j.watres.2008.07.020

[gwat13132-bib-0027] Rodriguez‐Escales, P. , A. Folch , G. Vidal‐Gavilan , and B.M. van Breukelen . 2016. Modeling biogeochemical processes and isotope fractionation of enhanced in situ biodenitrification in a fractured aquifer. Chemical Geology 425: 52–64.

[gwat13132-bib-0028] Rossum, G.V. 2017. Python 3.5.3. Python Software Foundation. http://www.python.org

[gwat13132-bib-0029] Smith, M.B. , A.M. Rocha , C.S. Smillie , S.W. Olesen , C. Paradis , L. Wu , J.H. Campbell , J.L. Fortney , T.L. Mehlhorn , K.A. Lowe , J.E. Earles , J. Phillips , S.M. Techtmann , D.C. Joyner , D.A. Elias , K.L. Bailey , R.A. Hurt Jr. , S.P. Preheim , M.C. Sanders , J. Yang , M.A. Mueller , S. Brooks , D.B. Watson , P. Zhang , Z. He , E.A. Dubinsky , P.D. Adams , A.P. Arkin , M.W. Fields , J. Zhou , E.J. Alm , and T.C. Hazen . 2015. Natural bacterial communities serve as quantitative geochemical biosensors. MBio 6, no. 3: e00326‐15. 25968645 10.1128/mBio.00326-15 PMC4436078

[gwat13132-bib-0030] Spain, A.M. , A.D. Peacock , J.D. Istok , M.S. Elshahed , F.Z. Najar , B.A. Roe , D.C. White , and L.R. Krumholz . 2007. Identification and isolation of a *Castellaniella* species important during biostimulation of an acidic nitrate‐ and uranium‐contaminated aquifer. Applied and Environmental Microbiology 73, no. 15: 4892–4904. 17557842 10.1128/AEM.00331-07 PMC1951013

[gwat13132-bib-0031] Techtmann, S.M. , J.L. Fortney , K.A. Ayers , D.C. Joyner , T.D. Linley , S.M. Pfiffner , and T.C. Hazen . 2015. The unique chemistry of eastern Mediterranean water masses selects for distinct microbial communities by depth. PLoS One 10, no. 3: e0120605. 25807542 10.1371/journal.pone.0120605 PMC4373936

[gwat13132-bib-0032] Vidal‐Gavilan, G. , R. Carrey , A. Solanas , and A. Soler . 2014. Feeding strategies for groundwater enhanced biodenitrification in an alluvial aquifer: Chemical, microbial and isotope assessment of a 1D flow‐through experiment. Science of the Total Environment 494: 241–251. 25051326 10.1016/j.scitotenv.2014.06.100

[gwat13132-bib-0033] Wang, Q. , G.M. Garrity , J.M. Tiedje , and J.R. Cole . 2007. Naive Bayesian classifier for rapid assignment of rRNA sequences into the new bacterial taxonomy. Applied and Environmental Microbiology 73, no. 16: 5261–5267. 17586664 10.1128/AEM.00062-07 PMC1950982

[gwat13132-bib-0034] Watson, D.B. , J.E. Kostka , M.W. Fields , and P.M. Jardine . 2004. The Oak Ridge Field Research Center Conceptual Model. Oak Ridge, Tennessee: United States Department of Energy.

[gwat13132-bib-0035] Wu, L.Y. , C.Q. Wen , Y.J. Qin , H.Q. Yin , Q.C. Tu , J.D. Van Nostrand , T. Yuan , M.T. Yuan , Y. Deng , and J.Z. Zhou . 2015. Phasing amplicon sequencing on Illumina Miseq for robust environmental microbial community analysis. BMC Microbiology 15: 125. 10.1186/s12866-015-0450-4 26084274 PMC4472414

[gwat13132-bib-0036] Wu, W.M. , J. Carley , J. Luo , M.A. Ginder‐Vogel , E. Cardenas , M.B. Leigh , C.C. Hwang , S.D. Kelly , C.M. Ruan , L.Y. Wu , J. Van Nostrand , T. Gentry , K. Lowe , T. Mehlhorn , S. Carroll , W.S. Luo , M.W. Fields , B.H. Gu , D. Watson , K.M. Kemner , T. Marsh , J. Tiedje , J.Z. Zhou , S. Fendorf , P.K. Kitanidis , P.M. Jardine , and C.S. Criddle . 2007. In situ bioreduction of uranium (VI) to submicromolar levels and reoxidation by dissolved oxygen. Environmental Science & Technology 41, no. 16: 5716–5723. 17874778 10.1021/es062657b

[gwat13132-bib-0037] Wu, W.M. , J. Carley , T. Gentry , M.A. Ginder‐Vogel , M. Fienen , T. Mehlhorn , H. Yan , S. Caroll , M.N. Pace , J. Nyman , J. Luo , M.E. Gentile , M.W. Fields , R.F. Hickey , B.H. Gu , D. Watson , O.A. Cirpka , J.Z. Zhou , S. Fendorf , P.K. Kitanidis , P.M. Jardine , and C.S. Criddle . 2006. Pilot‐scale in situ bioremedation of uranium in a highly contaminated aquifer. 2. Reduction of U(VI) and geochemical control of U(VI) bioavailability. Environmental Science & Technology 40, no. 12: 3986–3995. 16830572 10.1021/es051960u

